# Pdsg1 and Pdsg2, Novel Proteins Involved in Developmental Genome Remodelling in *Paramecium*

**DOI:** 10.1371/journal.pone.0112899

**Published:** 2014-11-14

**Authors:** Miroslav Arambasic, Pamela Y. Sandoval, Cristina Hoehener, Aditi Singh, Estienne C. Swart, Mariusz Nowacki

**Affiliations:** Institute of Cell Biology, University of Bern, Bern, Switzerland; Centre National de la Recherche Scientifique, France

## Abstract

The epigenetic influence of maternal cells on the development of their progeny has long been studied in various eukaryotes. Multicellular organisms usually provide their zygotes not only with nutrients but also with functional elements required for proper development, such as coding and non-coding RNAs. These maternally deposited RNAs exhibit a variety of functions, from regulating gene expression to assuring genome integrity. In ciliates, such as *Paramecium* these RNAs participate in the programming of large-scale genome reorganization during development, distinguishing germline-limited DNA, which is excised, from somatic-destined DNA. Only a handful of proteins playing roles in this process have been identified so far, including typical RNAi-derived factors such as Dicer-like and Piwi proteins. Here we report and characterize two novel proteins, Pdsg1 and Pdsg2 (*Paramecium protein involved in Development of the Somatic Genome 1 and 2)*, involved in *Paramecium* genome reorganization. We show that these proteins are necessary for the excision of germline-limited DNA during development and the survival of sexual progeny. Knockdown of *PDSG1* and *PDSG2* genes affects the populations of small RNAs known to be involved in the programming of DNA elimination (scanRNAs and iesRNAs) and chromatin modification patterns during development. Our results suggest an association between RNA-mediated trans-generational epigenetic signal and chromatin modifications in the process of *Paramecium* genome reorganization.

## Introduction

In ciliates, such as *Paramecium*, small RNAs participate in the programming of large-scale DNA deletion and genome organization during development. A characteristic feature of ciliates is the division of germline and somatic functions between two types of nuclei: the diploid micronucleus (MIC) and the polyploid macronucleus (MAC) respectively. New MIC and MAC develop from zygotic nuclei produced by fusion of haploid parental MIC during sexual cycle. In this process, a new MAC genome (72 Mb in *Paramecium tetraurelia*) matures from the MIC genome (97 Mb) by extensive editing [1] which is accompanied by polyploidization to an average copy number of ∼800n. Genome correcting during development discards most non-genic DNA, transposable elements and other repeated sequences and numerous Internal Eliminated Sequences (IESs). While the new MAC is developing, the old MAC is fragmented and later eliminated from the cell.

*Paramecium tetraurelia* IESs are abundant (∼45,000), short (frequently less than 30 bp long), single-copy, noncoding sequences that are precisely excised from MIC DNA to produce the mature MAC genome [1]. Each *Paramecium* IES is flanked by two 5′-TA-3′ dinucleotides (TA repeats). IES excision leads to the retention of a single TA dinucleotide. The ends of IESs have symmetrical inverted base frequencies, which can be crudely represented by the consensus sequence TAYAGYNR with no other known conserved motifs. IES ends are similar to the ends of Tc1/mariner transposons [2] but this consensus is recognized by the *Paramecium* IES excisase, a domesticated PiggyBac-related transposase (PiggyMac)[3]. The IES end sequence also appears to be important for the staggered double-strand cuts that initiate excision [4]–[6].

In *Paramecium*, both precise excision of IESs and imprecise elimination of genomic regions containing transposable elements can be controlled by maternal effects. This epigenetic dependency was demonstrated by microinjection of DNA, in the form of an IES sequence. The introduction of an IES into the old MAC can prevent the elimination of identical sequences from the progeny's somatic genome [7], [8]. However; IESs are retained to different degrees depending on the quantity of injected sequence and the sensitivity of the IES. The retention is inherited in future generations and is true for a third of total *Paramecium* IESs assayed [8], known as maternally controlled IESs, or mcIESs.

These observations imply that trans-nuclear genome comparison occurs during development.

In ciliates, multiple RNA interference-related pathways exist. Post-transcriptional gene silencing can be induced by untranslatable transgenes [9], [10] or by feeding cells with *E. coli* producing double-stranded RNAs [11]. In both cases, silencing of targeted genes generates complementary ∼23 nt siRNAs produced by Dicer-related protein (Dcr1) [12]–[15]. In *Paramecium tetraurelia* and the related Oligohymenophorean ciliate *Tetrahymena thermophila*, a second RNAi-related pathway employs a distinctive class of sRNAs known as "scan RNAs" (scnRNAs) to perform the trans-nuclear comparison for the precise targeting of DNA elimination. In *Paramecium* and *Tetrahymena* Dicer-like proteins are responsible for producing scnRNAs in the meiotic MIC and Piwi proteins for binding and protecting scnRNAs [14], [16]–[19]. High throughput sequencing of *Paramecium* sRNA has demonstrated that scnRNAs initially correspond to the entire germline genome, and become progressively enriched in IESs matching sequences [20], [21]. This enrichment of IESs matching sequences is the result of the reduction of the total population of scnRNA and proposed to be due to a process known as "RNA scanning". scnRNAs produced from transcripts across the MIC genome are filtered by pairing to transcripts from the old MAC genome leaving only scnRNAs matching to the germline-limited sequences [14], [22]–[24]. The remaining germline-specific scnRNAs are then transported to the developing new MAC where they target DNA elimination [25]. In *Paramecium* scnRNAs are produced by a pair of paralogous Dicer-like proteins (Dcl2 and Dcl3) [14] and become associated with a pair of Piwi-like protein paralogs (Ptiwi01p and Ptiwi09p) [16]. The mechanism by which scnRNAs trigger DNA elimination in the developing MAC is not completely understood. In *Tetrahymena* scnRNAs are responsible for trimethylation of lysine 27 and lysine 9 of histone H3 (H3K27me3 and H3K9me3 respectively) within chromatin destined to be eliminated [26]–[28]. It is therefore possible that scnRNA-mediated chromatin modification defines genomic regions to be targeted by DNA excision machinery.

In *Paramecium*, a third class of development-specific sRNAs is present, iesRNAs varies in length from ∼21–31 nt (peaking at 27/28 nt) and are produced in the new MAC by a distinct Dicer-like protein, Dcl5 [21]. iesRNAs appear at late stages of development when IESs are being excised and match IESs exclusively, leading to the proposal that they may be derived from excised IESs [21]. Silencing of *DCL5* shows that iesRNAs are involved in targeting DNA excision in the developing MAC [21].

In *Paramecium* one of the key players in the RNA-mediated trans-nuclear crosstalk is the Nowa1 protein (Nowa1) [15]. Nowa1 is expressed specifically during sexual development and is required for the elimination of transposons and maternally controlled IESs [15]. It accumulates in the maternal MAC shortly before meiosis and later translocates to the developing MAC. The functions of Nowa1 in both maternal MAC and developing MAC are still under examination.

In this study we report the discovery of two proteins, Pdsg1 and Pdsg2, involved in *Paramecium* genome development. Both proteins are necessary for the excision of germline-limited DNA and for the survival of sexual progeny. Through high-throughput sequencing we show that *PDSG1* and *PDSG2* knockdowns affect *Paramecium'*s development-specific sRNAs. Together these results suggest that these proteins are involved in the epigenetic programming of DNA remodelling and in IES excision. In addition, the knockdowns affect chromatin modification patterns during development which suggests a link between DNA elimination and histone modifications in the process of *Paramecium* genome maturation.

## Materials and Methods

### *Paramecium* cultivation

*Paramecium* strain*51*, mating type 7 was used in all experiments. *Paramecium* cells were cultured in Wheat Grass Powder (WGP; Pines International, Lawrence, KS) medium bacterized with *Klebsiella pneumoniae*, and supplemented with 0.8 mg/l of β-sitosterol. Cultures used in all the experiments were grown at 27°C.

### Silencing experiments, survival test and IES retention PCR

For the silencing constructs different regions of the coding sequences of each candidate were selected and cloned into L4440 plasmid (list of specific primers can be found in Fig. S1). The plasmid was used for the transformation of HT1115 (DE3) *E.coli* strain. *Paramecium* cells were seeded into silencing medium at a density of 200 cells/ml and silencing was carried out as previously described [29]. Upon completion of development, single cells (n = 30) were isolated in fresh medium for the evaluation of survival of the progeny. Cells were monitored for 12 cell cycles after their isolation and categorized into three groups according to the observed phenotype (normal, sick or unviable). In parallel, 50 ml cultures were harvested and DNA extraction was performed by using GeneElute – Mammalian Genomic DNA MIniprep Kit (Sigma-Aldrich). IES PCR was done with GoTaq polymerase (Promega) standard protocols.

### Dot blot

Dot blot assays were conducted following standard protocols [30]. In detail, 3 µg of DNA of post-developmental cultures were fixed on a nylon membrane. Sardine and Thon transposons specific probes were labelled with α-32P dATP (3000 Ci/mmol) using RadPrime DNA Labeling System (Invitrogen). Probe against *ACTIN* gene targets the first 240 bp of the coding sequence. The signal was quantified with ImageJ 1.48e.

### Northern blot

10 µg of RNA were separated by electrophoresis in a denaturing gel and transferred to a nylon membrane. Full-length *PDSG1* gene probes were used for the specific detection. Probe against *PDSG2* targeted the 960 bp region, the same region that was used to clone the silencing fragment.

### GFP tagging, microinjection and GFP localization experiment

A set of specific primers (5′-TGATTTACAATTAAGGATTAGGAGTATTTTGA-3′ and (5′-CAGGCATTGATTGTATTTTAATTAATTTTAAATCT-3′) were used for the amplification of *PDSG1* gene including 175 bp upstream and 68 bp downstream of the coding region. Full length *PDSG2* gene along with 414 bp upstream and 343 bp downstream was amplified (5′-CGATAAAAGTTTGTTTTAATAAAATGATAATAAATCTCATAAAAGTG-3′ and 5′-GTATTTACTGCAGGTTTTTTTTGAATTGCATAAAC-3′). In case of *PDSG2*, GFP was inserted at the N-terminus. *PDSG1* was tagged at both ends but only the C-terminal tagged version was expressing a functional GFP. The constructs were linearized and microinjected into the MAC of the vegetative cells. Positively injected clones were selected by dot blot analysis. Cells were collected at different time points during sexual development and counterstained with DAPI (4,6-diamidino-2-2phenylindole). Images for this experiment were acquired with a Leica microscope (Wetzlar, Germany).

### Immunocytochemistry and confocal microscopy

Cells were collected during sexual development and prepared for immunostaining according to standard protocol [31]. Anti-trimethyl-Histone H3 (Lys9) antibody (07-442, Millipore) and Anti-trimethyl-Histone H3 (Lys27) (07-449, Millipore) at 1∶100 dilution were used. A FLUOVIEW FV1000 (Olympus) system with PLAPON 60× O SC NA 1.40 objective was used for imaging capture.

### Peptide competition assay

Total protein extract form an intermediate developmental stage was loaded in triplicate and proteins were separated by SDS-PAGE. Prior to immunoblotting, 0.66 ng of Anti-trimethyl-Histone H3 (Lys27) was diluted 1∶3000 in 5% BSA supplemented with 0.1% Tween-20. Diluted antibody was incubated with dilution buffer only or with 0.02 ug of unmodified human histone H3 peptide (Ab2903, Abcam) or with 0.02 ug of human histone H3 (tri methyl K27) peptide (Ab1782, Abcam) for 3 h at the room temperature. Blot was incubated with secondary HRP conjugated antibody (Sc-2004, Santa Cruz Biotechnology).

### Small RNA analysis

*Paramecium* cells from 800 ml of culture were harvested and resuspended in 6 ml of TRI reagent BD (Sigma-Aldrich). Total RNA extraction was carried out following the TRI Reagent BD protocol. The enrichment of small RNA was performed using mirVana miRNA Isolation Kit (Ambion). Enriched samples were used for library preparation following the TruSeq Small RNA Sample Preparation protocol (Illumina). Reads were mapped with BWA [32] and uniquely mapping sRNAs selected by a custom Python script. To generate the sRNA size histograms, we normalized the number of sRNAs by the total number of mapped sRNAs (both MAC genome- and IES-matching).

### Accession Numbers

*PDSG1* and *PDSG2* sequences are available from the GenBank under the accessions: XM_001442856 and XM_001425883, respectively. *ACTIN* gene accession number: XM_001443584. Raw sRNA sequence data for the *PDSG1*-KD, *PDSG2*-KD, control early and control late time points can be obtained from the European Nucleotide Archive (ENA) under the accessions: PRJEB5853, PRJEB5867, SRR907874 and SRR907875, respectively.

### Protein domain prediction

Homology detection and structure prediction were estimated by open access Pfam (http://pfam.sanger.ac.uk/search) [33] and HHpred (toolkit.tuebingen.mpg.de/hhpred)[34] software.

## Results

### Selection of candidate genes involved in genome development in *Paramecium tetraurelia*

Only a handful of key factors playing role in *Paramecium* genome development have been described so far [3], [15], [16], [21], [35]. A common feature of all these genes is the transcriptional upregulation during sexual reproduction. We took this characteristic transcriptional upregulation as the main criterion for the selection of putative factors involved in MAC development and more specifically in the RNA-mediated genome reorganization.

A publicly accessible BioMart database [36] was used to query microarray data during the life cycle of *Paramecium* [37]. We chose twenty-eight genes that are highly upregulated during MAC development, all of which have no identifiable paralogs in the *Paramecium* genome (Fig. S2). The selected candidate genes were subdivided into two groups according to their expression profiles. Twelve candidates are early expressed genes and the peak of their expression is during meiosis of MIC or MAC fragmentation (Fig. S3A). The second group of 16 candidates included late expressed genes whose maximum expression occurs during the formation of the new MAC (Fig. S3B). Among the 28 selected candidates, the proteins of 10 contain predicted domains while the rest of the candidates have no homology to known proteins.

### Pdsg1 and Pdsg2 are essential for the generation of sexual progeny

To determine whether the developmentally upregulated gene candidates have an effect on the formation of fully functional progeny; we silenced each one of the genes independently during development and observed the effects on the offspring based on the ability of maintaining normal vegetative growth. The silencing was induced by feeding *Paramecium* cells with *E. coli* expressing dsRNA corresponding to the target genes (see Materials and Methods and supplementary information). *E. coli* producing dsRNA corresponding to the empty bacterial plasmid was used as negative control (empty vector control (EV)) and as a positive control we used a NOWA1 silencing construct which blocks the excision of maternally controlled IESs and is lethal to the sexual progeny [15].

**Figure 1:**
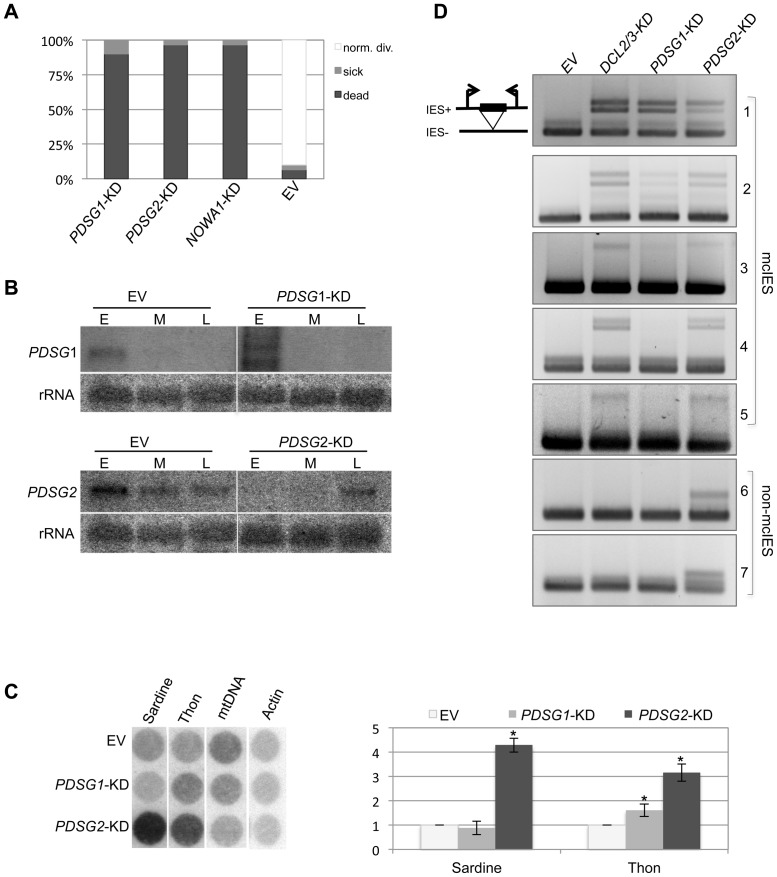
Effects of *PDSG1* and *PDSG2* silencing on progeny survival and IES excision. (A) Survival test. Graphic representation of percent of normally dividing (white), sick (grey) and dead (black) progeny cells. The silencing of *PDSG1* and *PDSG2* was lethal in 95% and 97% of cells, respectively. *NOWA1*-KD is a positive control. Empty vector (EV) is a negative control. (B) *PDSG1* and *PDSG2* silencing efficiency was assayed by Northern blot by comparing the control (EV, empty vector) and silenced cultures. Three developmental points were analysed during sexual cycles. Early developmental stage (E) includes cells undergoing meiosis and 50% of cells present fragmented old MAC. Middle developmental stage (M) presents 100% of the cells with fragmented old MAC. Late developmental stage (L) includes cells with fragmented old MAC and a substantial number of cells with evident new developing MAC. (C) Effect of *PDSG1* and *PDSG2* silencing on transposon elimination. Macronulcear DNA was extracted form *PDSG1*-KD and *PDSG2*-KD cultures and analyzed for the retention of Sardine and Thon transposons using specific probes. Quantification signal of two classes of transposons was normalized to the mitochondrial DNA probe (mtDNA). * A two-tailed Student's t-test was used to assess statistical significance of the differences in the mean (values of bars), and an asterisk is shown if the p-value from this test is <0.05. For Sardine elements p-values are: PDSG1-KD vs Ctrl: 0.054; PDSG2-KD vs Ctrl: 2.7e-3. For Thon elements p-values are: PDSG1-KD vs Ctrl: 8.8e-5; PDSG2-KD vs Ctrl: 2.3e-4. Probe against Actin gene was used as an additional loading control. (D) IES retention PCR. Excision of 5 mcIES (1–5) and 2 non-mcIESs (6–7) are shown. Upper band represents IES+, lower band represents DNA with excised IES (IES-). IES: 1 (mtA promoter IES); 2 (51G4404); 3 (51A6649); 4 (51A2591); 5 (51G2832); 6 (51G1413); 7 (51A1835).

The silencing of each of the 28 candidate genes was started 3 to 4 vegetative cell cycles before commitment to development. None of the silencing had a noticeable effect on cell growth during the vegetative cycle. To assess the survival of the sexual progeny, from each of the experiments 30 random post-developmental cells were monitored individually and scored for survival. Among the twenty-eight candidates only two showed impaired cell viability (Fig 1A). We cannot exclude the possibility that some of the remaining 26 candidates are also involved in developmentally specific processes, since the efficiency of each RNAi silencing construct has not been determined. The two genes essential for the generation of sexual progeny were named as *Paramecium protein involved in Development of the Somatic Genome 1 and 2* ( *PDSG1* and *PDSG2*). The dramatic effect on the survival of sexual progeny of *PDSG1*-KD and *PDSG2*-KD (90% and 93%) was comparable to Nowa1 depletion (used as control), suggesting that Pdsg1 and Pdsg2 are essential for adequate completion of developmental process in *Paramecium*. In addition to the survival test, silencing efficiency was assessed by Northern blot (Fig. 1B). Furthermore, no evident delay in the progression of developmental stages was noticed after cytological evaluation of major structures (MIC, old MAC and new MAC) in PDSG1, and PDSG2 silenced cultures. All the silencings were repeated 5 times and were highly reproducible (data not shown).

### Depletions of Pdsg1 and Pdsg2 impair elimination of germline-limited DNA in the developing macronucleus

In order to determine whether Pdsg1and Pdsg2 were involved in the *Paramecium* DNA elimination process we checked for retention of MIC-limited sequences in the new MAC genome upon the completion of development. As mentioned earlier, precise and imprecise mechanisms of DNA elimination determine the genome content in the new MAC. Both mechanisms may include common set of factors but some of them may be unique in order to determine the precision of the elimination that differ between both mechanisms.

Since repetitive sequences like minisatellites and transposable elements are imprecisely eliminated from the new MAC [38], we checked for the correct removal of Sardine and Thon transposons. The level of retention of these transposons was measured in MAC genomic DNA samples collected from cells that have completed sexual development (progeny) from control (EV), *PDSG1*-KD, and *PDSG2*-KD cultures (Fig. 1C). Hybridization with specifics probes showed that the silencing of *PDSG1* has slight effect on the elimination of Thon of transposons but not on the elimination of Sardine since the levels of retention were similar to the levels observed in the control. However, depletion of Pdsg2 induces a strong retention of Sardine and Thon transposons. These results were corroborated by two independent experiments and suggest that only Pdsg2 is involved in the imprecise mechanism of DNA elimination.

Next, the precise elimination of IESs in the new MAC was assayed by PCR on total genomic DNA from cells that have completed development. We tested 7 different IESs; 5 maternally and 2 non-maternally controlled, with primers located in DNA regions flanking IESs (Fig. 1D and Fig. S4). Dcl2/3 silenced cells were used as a positive control for the retention of maternally controlled IESs. The depletion of both, *PDSG1*, and *PDSG2*, prevents the accurate excision of maternally controlled IESs (Fig. 1D, 1–5). *PDSG2* silencing seems to have a stronger effect in the retention of this group of IESs than *PDSG1*silencing since the later only affects three of the maternally controlled IESs. Furthermore, only the silencing of *PDSG2* affects the excision of non-maternally controlled IESs. This finding identifies *PDSG2* as one of the few known factors involved in a general mechanism of DNA elimination [39], [40]. These results were reproduced in four independent experiments and are summarized in Fig. S5.

**Figure 2:**
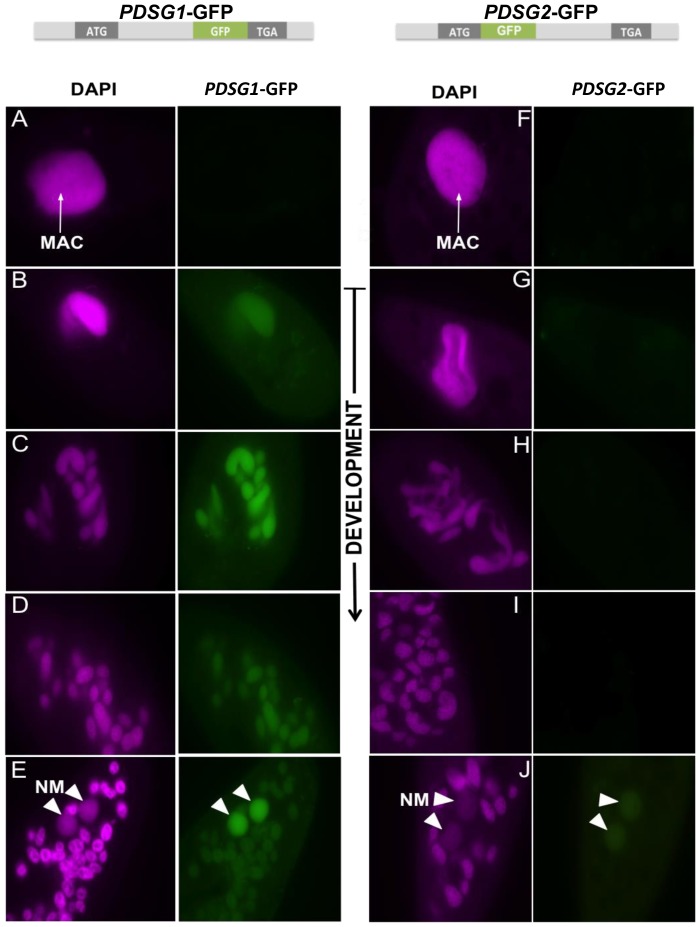
Subcellular localization of Pdsg1-GFP and Pdsg2-GFP fusion proteins during development. 1. Graphical representation of C-terminally tagged *PDSG1* with GFP. 2. Graphical representation of N-terminally tagged *PDSG2* with GFP. (A–E) Localization pattern of PDSG1-GFP. (F–J) Localization pattern of Pdsg2-GFP. (A, F) Vegetative cells with the intact MAC. (B, C, G, H) beginning of old MAC fragmentation that represents early development. (D, I) Middle stage of development with fragmented MAC. (E, J) Late development when new MAC is formed while the fragments of the old MAC are still present in the cytoplasm. Magenta: DAPI; green: GFP; white arrow: old macronucleus; arrowheads: new MAC (NM).

**Figure 3:**
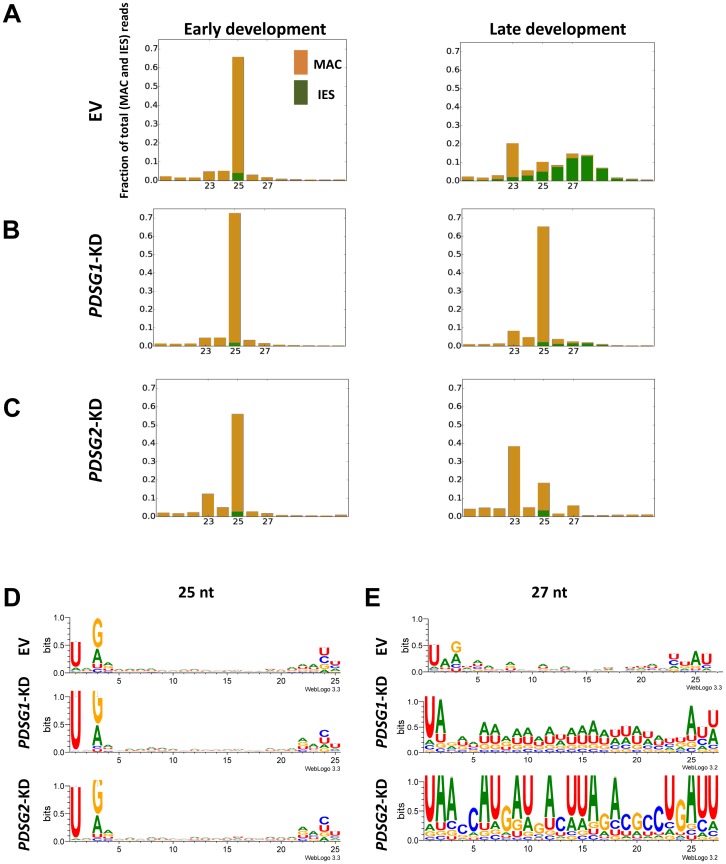
Effects of *PDSG1* and *PDSG2* knockdowns on developmental-specific small RNA. (A–C) Histograms of sRNAs size for MAC genome-matching (yellow) and IES- matching (green) reads of the early and late developmental stages for control and *PDSG1* and *PDSG2* knockdowns. (D, E) Representative sequence logos of 25 nt and 27 nt sRNAs for control and knockdown cells from the late developmental stage. Note that due to the low abundance of iesRNAs in the knockdowns in (E) a single sRNA is relatively abundant in the 27 nt sRNAs.

**Figure 4:**
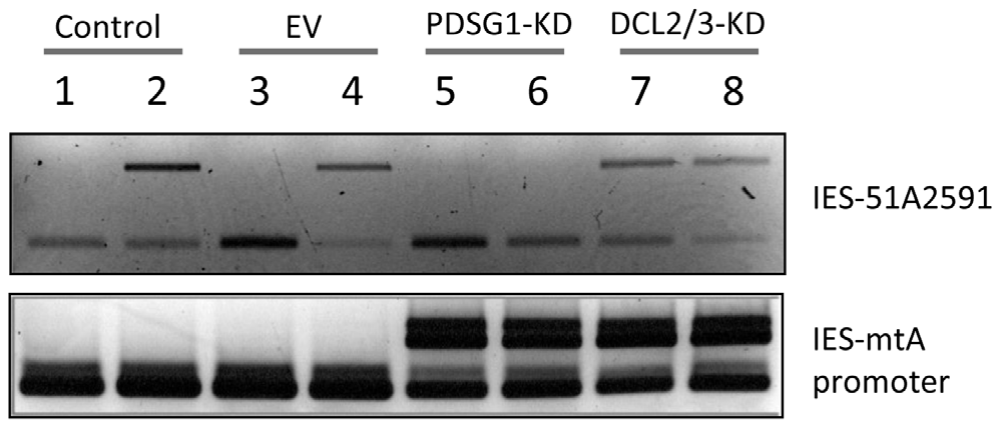
Effect of PDSG1 depletion on the retention of injected IES (51A2591). Lanes 1 and 2, control non-silenced cells. Lanes 3 and 4, control silencing with empty vector (EV). Lanes 5 and 6, PDSG1 depleted cells. Lanes 7 and 8, DCL2 and DCL3 depleted cells. Lanes 2, 4, 6 and 8, IES 51A2591 injected cells. IES-mtA promoter (control mcIES).

**Figure 5:**
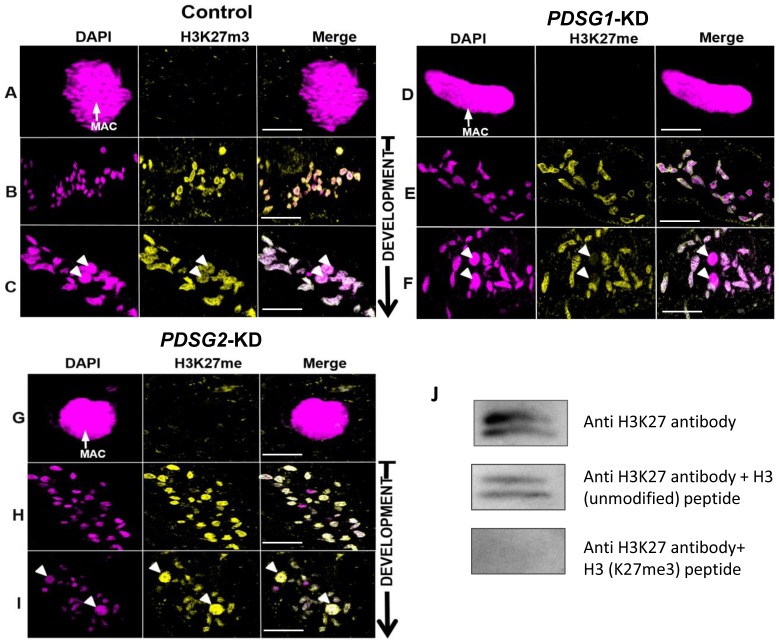
Effects of Pdsg1 and Pdsg2 depletion on H3K27me3 histone modification. (A, D, G) Maternal MAC in vegetative cells of control, *PDSG1*-KD and *PDSG2*-KD. (B, E, H) Middle stage of development with the fragments decorated with H3K27me3. (C, F, I) Late stage of development with histone modification present in the new MAC. J Peptide competition assay. Magenta: DAPI; yellow: H3K27me3; white arrow: MAC; arrowhead: new MAC. Scale bar 5 µm.

### Pdsg1 is present in both maternal and developing nuclei whereas Pdsg2 localizes exclusively to developing MAC

GFP fusion proteins were generated in order to monitor the subcellular localization of Pdsg1 and Pdsg2 during development (Fig. 2). The expression of Pdsg1-GFP and Pdsg2-GFP was undetectable during vegetative growth (Fig. 2A, F). Furthermore, both Pdsg1-GFP and Pdsg2-GFP signals were exclusively detected during development.

Pdsg1-GFP was detected in the parental MAC during early development (Fig. 2B) and the signal was persistent throughout development showing a dynamic localization from the parental MAC to the newly developing MAC (Fig. 2C, D, E). The Pdsg1-GFP signal gained intensity in the new MAC at late developmental stages. On the other hand, Pdsg2-GFP signal was not detected in the cells at early developmental stages and only appeared in the new MAC (Fig. 2G, H, I, J). Both transgenes do not alter either cell viability or the process of DNA elimination, which was confirmed by the survival test and IES retention PCRs, respectively (data not shown).

### *PDSG1* knockdown blocks RNA scanning process while *PDSG2* knockdown affects iesRNA population

To investigate possible effects of *PDSG1* and *PDSG2* knockdowns on developmental-specific small RNA, we examined sRNA-seq data from *PDSG1* and *PDSG2*-silenced cells. Early and late developmental stages were analyzed (correspond to the stages described in Fig. 1B). In cells fed with empty vector (EV, control), 25 nt sRNAs are mostly composed of scnRNAs (based on the high proportion of 5′UNG molecules [21] (Fig. 3D). As previously shown by absolute quantification of sRNAs from electrophoretic gels, there is a significant decrease in quantity of scnRNAs in the late developmental stage due to the elimination of MAC genome-matching scnRNAs during the scanning process (Fig. 3A). In *PDSG1*-KD cells the elimination of 25 nt scnRNAs was suppressed (Fig. 3B), indicating that the scanning process is altered. This suggests that *PDSG1* is involved, indirectly or directly, either in the transport of scnRNA into the parental MAC, facilitating the interaction between scnRNAs and their targets in the parental MAC, or in the elimination of MAC genome-matching scnRNAs. In the case of *PDSG2* knockdown, no aberrant effect on scnRNA quantity was observed (Fig. 3C).

iesRNAs (peaking at 27–28 nt) appear in the late developmental stage in cells fed with empty vector (control) (Fig. 3A) as a consequence of Dcl5 cleavage of RNA which is likely transcribed from excised DNA [21]. This assumption is based on the fact that all iesRNAs map precisely to IES sequences with none of them overlapping MAC-IES junctions. In addition, the fact that iesRNAs originate only from excised IESs and not from non-excised IESs in the case of knockdown of proteins that are involved in IES excision but affect only a fraction of IESs. The inhibition of the scanning process in the parental MAC during the depletion of *PDSG1* prevents the normal excision of IESs during development. The low amount of iesRNAs seen in sRNA histograms of *PDSG1*-KD in late development (Fig. 3B) may thus be a direct consequence of a higher relative quantity of scnRNAs in these samples as well as massive IES retention that would prevent the production of iesRNAs. Depletion of *PDSG2* does not appear to inhibit RNA scanning (MAC genome-matching scnRNAs are reduced), but does prevent the production of iesRNAs at late developmental stage (Fig. 3C). The inhibition of iesRNAs can either be due to a complete retention of all IESs or due to a more direct involvement of Pdsg2 in iesRNA production.

### Pdsg1 is directly involved in the scanning process

In order to determine the role of Pdsg1 in the scanning process we monitored the retention of a specific IES (51A2591) during the depletion of Pdsg1. In wild type strain the IES-51A2591 is normally excised after development and its excision was not affected in PDSG1-KD strains (Fig. 1D, IES number 4). First, we challenged the system by injecting the IES-51A2591 into the maternal MAC, which will promote the retention of this specific sequence in the progeny MAC, as previously reported [8] (Fig. 4, lane 2). As a control, and to monitor the normal progression of development, both strains were subjected to feeding with *E. coli* producing RNA from empty vector construct (Fig. 4, lanes 3 and 4). Since empty vector control does not include target sequences for silencing, in the progeny of the wild type background IES-51A2591 was correctly excised (Fig. 4, lane 3). Conversely, under this condition in the injected strain the retention of IES-51A2591 was passed to the progeny as anticipated (Fig. 4, lane 4). Next, *PDSG1* was silenced in wild type strain and compared to the IES-51A2591 injected strain (Fig. 4, lanes 5 and 6 respectively). As expected, the depletion of Pdsg1 in wild type background did not promote the retention of IES-51A2591 (Fig. 4, lane 5). However, the silencing of *PDSG1* promoted complete excision of the IES-51A2591 in the injected strain (Fig. 4, lane 6). This suggests that the depletion of Pdsg1 has a negative effect on the scanning process, arguing that Pdsg1has a direct functional role in the scanning. Furthermore, elimination of the scnRNA production (Dcl2/3 silencing, Fig. 4, lanes 7 and 8) was used as a positive control of the disruption of the scanning process. In this case IES-51A2591 was retained in both strains due to the absence of scanning molecules (scnRNAs).

### Depletion of Pdsg2 affects the distribution of Dcl5 in the new MAC

Since both *PDSG1-* and *PDSG2*-KD affect the production of iesRNAs we decide to check the consequences of the depletion of Pdsg1 and Pdsg2 on Dcl5 localization. As previously described [21], Dcl5-GFP fusion protein in wild type cells forms distinct foci in the developing MACs (Fig. S6). The depletion of Pdsg1 had no obvious effect on the normal expression or the foci formation of Dcl5. In contrast, the depletion of Pdsg2 disrupts Dcl5 foci organization. Although Dcl5 is still expressed only in the new MAC the number and size of Dcl5 foci is completely altered.

### Knockdowns of *PDSG1* and *PDSG2* affect histone methylation during development

Since H3K27me3 and H3K9me3 histone marks are associated with DNA elimination in *Tetrahymena* we decided to test if these decorations are also present in *Paramecium* and whether they are affected by the knockdowns of *PDSG1* and *PDSG2*.

Similar to *Tetrahymena*, both H3K27me3 and H3K9me3 are undetectable in the macronucleus of vegetative cells [26], [28] (Fig. 5A, D, G and Fig. S7A, D and G). In cells fed with empty vector control, the staining for H3K27me3 became evident at early stages of development (Fig. 5B) where it is present in the fragments of the old MAC. At later developmental stages, an H3K27me3 signal was also evident in the developing MAC (Fig. 5C) where the signal intensity was comparable to the one in the fragmented parental MAC. The H3K27me3 signal in the fragments of the parental MAC was slightly weaker in *PDSG1*-KD cells (Fig. 5E) when compared to cells fed with empty vector (control), but it was completely absent in the developing MACs (Fig. 5F). The lack of H3K27me3 in the developing MAC of *PDSG1*-KD cells suggests a link between RNA scanning in the parental MAC and histone modification in the developing MAC, both being required for epigenetic programming of the elimination of maternally controlled IESs.

The H3K27me3 signal in the fragments of the parental MAC was not removed by depletion of *PDSG2* (Fig. 5H). However, these cells had a considerably stronger H3K27me3 signal in the developing MACs (Fig. 5I). This may suggest an accumulation of H3K27me3-marked chromatin due to a complete block of DNA elimination caused by the silencing of *PDSG2*. Furthermore, similar result was obtained with H3K9me3-specific antibody (Fig. S7).

H3K27me3 antibody specificity was confirmed by a peptide competition assay (Fig. 5J). Only in case when antibody was pre-incubated with H3K27me3 peptide, specific band was not detectible due to a complete out-competition of the antibody.

## Discussion

In *Paramecium* sRNAs guide the elimination of germline-limited sequences during development. scnRNAs, that derive from the germline genome, target the elimination of DNA sequences subject to epigenetic control from developing MAC during its development[41]. A second class of sRNAs, iesRNAs, produced from excised germline-specific DNA in the new MAC complements scnRNAs and ensures complete DNA excision [21]. In this study we identify and characterize two novel proteins in *Paramecium* that affect both DNA excision and the development-specific sRNA populations during new MAC genome reorganization.

**Figure 6:**
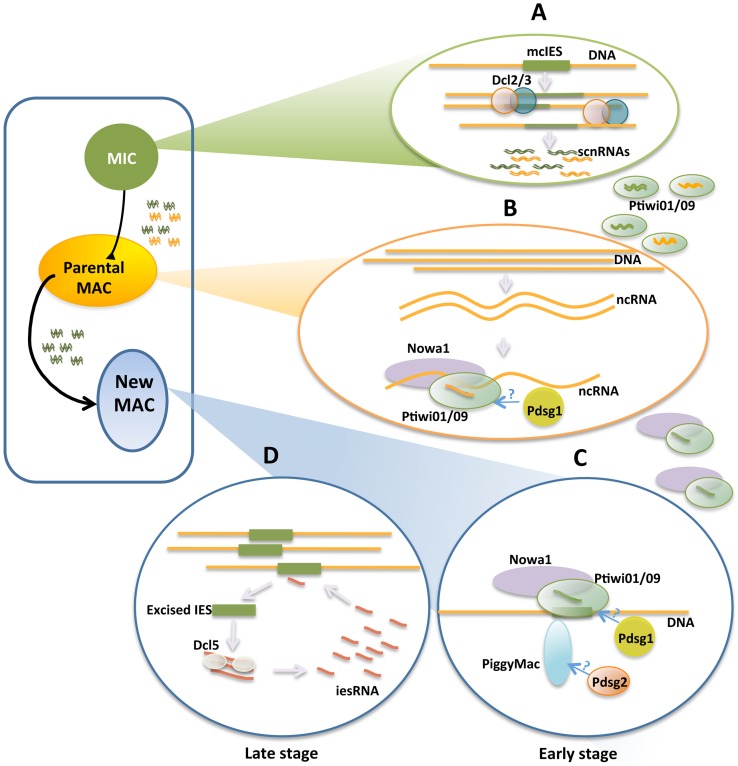
Proposed model of Pdsg1 and Pdsg2 roles in elimination of mcIESs during MAC development. (A) Early development, MIC germline genome is transcribed and transcripts are processed by Dcl2/3 into scnRNAs. (B) scnRNAs are transported from the MIC to parental MAC by Ptiwi01/09. Once in the parental MAC, scanning takes place filtering out the MAC genome-matching scnRNAs. We propose that the matching of scnRNAs to complementary sequences may be driven by a multiprotein complex that may include Pdsg1. (C) scnRNAs without matching sequences are transported to the new MAC where they target the excision of complementary sequences. This study suggests that Pdsg2 may be part of the DNA excision machinery. (D) After the excision, IESs are used as templates for iesRNA production to ensure reinforce the signal for an efficient targeting of IES excision.

*PDSG1* reaches the highest level of expression at the beginning of sexual development and remains expressed at a high level until the new MAC is formed. The localization of *PDSG1* is very similar to that of Nowa1 protein, which was shown to be involved in the excision of maternally-controlled IESs [15]. Nowa1 is an Argonaute and RNA binding domain-containing protein [15], and its putative role is to facilitate the interactions between Piwi-bound small RNAs and their targets in the maternal MAC (RNA scanning process) and in the developing MAC (targeting DNA excision).We show that depletion of *PDSG1* also blocks the excision of some maternally-controlled IESs, which may suggest that both Pdsg1 and Nowa1 are components of the same molecular machinery (Fig. 6). Surprisingly, the silencing of *PDSG1* does not affect the elimination of Sardine and Thon transposons which is strongly affected by *NOWA1* silencing [15]. No other protein in *Paramecium* is known to affect only the excision of mcIESs but not the transposons. This difference may indicate that the machinery involved in the process of marking transposons for elimination differs from the one employed in the selection of mcIESs even though the two germline-specific DNA elements seem to require sRNA machinery for their recognition and/or excision.

Our results suggest that Pdsg1 may be involved in scnRNA transport between nuclei or in the interaction of scnRNAs with the complementary target sequences (Fig. 6). Pdsg1 depletion prevents the degradation of MAC-matching scnRNAs and induces a massive accumulation of these sRNAs suggesting a direct role in the scanning process. In addition, we show that *PDSG1* knockdown prevents the retention of an IES that is normally retained in the developing MAC after microinjection of its sequence into the maternal MAC prior development (Fig. 4). Because retention of mcIESs is believed to be due to the sequestration of scnRNAs complementary to the injected IES in the parental MAC, our data suggests that Pdsg1 is involved in the scanning process. The reduced amount of iesRNAs in Pdsg1 knockdown at late developmental stage may be attributed to the reduced amount of excised IESs, which are prerequisites for transcription and iesRNA production.

Contrary to the effects of *PDSG1* knockdown, silencing of *PDSG2* does not perturb scnRNA quantities or the scanning. However, the production of iesRNAs is highly impaired by the depletion of Pdsg2 suggesting that iesRNA production is blocked because of the absence of excised IESs or that this protein might be involved in iesRNA production or stability (Fig. 6). The fact that Pdsg2 depletion affects the localization of Dcl5 may support the former idea. Furthermore, the substantial retention of mc- and non-mcIESs and the lethality of the progeny on PDSG2-KD indicate that Pdsg2 is essential for excision of IESs.

At the chromatin level, the methylation of histone H3 (H3K9me3 and H3K27me3), shown to be a necessary for scnRNA-dependent DNA elimination in *Tetrahymena* [26], is also present in *Paramecium*. In *Paramecium*, only the IESs that require sRNAs for excision may be dependent on histone modification since not all IESs require scnRNAs and iesRNAs for their excision [42]. This suggests that both ciliates present similar mechanism for targeting of DNA excision, however, as debated previously, in *Paramecium* this seems unlikely since some IESs are shorter than the length of DNA wrapped around a nucleosome [41]. The fact that *PDSG1* knockdown blocks the selection of scnRNAs, affects the methylation of histones and blocks the excision of maternally controlled IESs, may suggest that the these processes are associated with each other, but the precise mechanism still needs to be resolved. From the effects of the *PDSG2* knockdown it can be concluded that a block of IES excision induces an accumulation of H3K27me3 and H3K9me3. A future logical follow up to this study will be to determine potential binding partners of Pdsg1 and Pdsg2 to further elucidate their role in DNA elimination in *Paramecium*.

## Supporting Information

Figure S1 **Primers used for amplification of silencing constructs for all selected candidates.**
(TIF)

Figure S2 **Twenty-eight selected candidates with accession numbers and predicted protein domains.**
(TIF)

Figure S3 **Gene expression profiles of selected candidates.**
(TIF)

Figure S4 **Primers used for IES retention PCR.**
(TIF)

Figure S5 **Summarized results of IES retention PCR.** Seven tested IES are listed in the first column. IES retention is indicated with (+). Maternally controlled IESs are labelled as ‘mc’ and non-maternally controlled IESs as ‘non-mc’.
(TIF)

Figure S6 **Depletion of Pdsg2 affect the normal distribution of Dcl5-GFP in the new MAC.** Localization of Dcl5-GFP to the new MAC in control, PDSG1-KD and PDSG2-KD strains. DAPI staining (blue, DNA), Dcl5-GFP (green) and merge images are shown. White arrows indicate developing MACs. (bottom panel) New MAC is shown in detail from the merge images.
(TIF)

Figure S7 **The effects of** ***PDSG1*** **and** ***PDSG2*** **knockdowns on H3K9me3 histone modification.** (A, D, G) Maternal MAC in vegetative cells of EV, *PDSG1*-KD and *PDSG2*-KD. (B, E, H) Middle stage of development with the fragments decorated with H3K27me3. (C, F, I) Late stage of development with histone modification present in new MAC. Magenta: DAPI; yellow: H3K27me3; white arrow: MAC; arrow head: new MAC. Scale bar 5 µm.
(TIF)
