# Correction: Pdsg1 and Pdsg2, Novel Proteins Involved in Developmental Genome Remodelling in *Paramecium*

**DOI:** 10.1371/journal.pone.0118384

**Published:** 2015-02-25

**Authors:** The PLOS ONE Staff

In the Author Contributions section, Pamela Y. Sandoval (PS) should be listed as one of the persons who wrote the paper.
