# Supplementary figures and images for: Pdsg1 and Pdsg2, Novel Proteins Involved in Developmental Genome Remodelling in Paramecium
Source: PLoS One. 2014 Nov 14;9(11):e112899. doi: 10.1371/journal.pone.0112899 (PMC4232520; doi:10.1371/journal.pone.0112899)

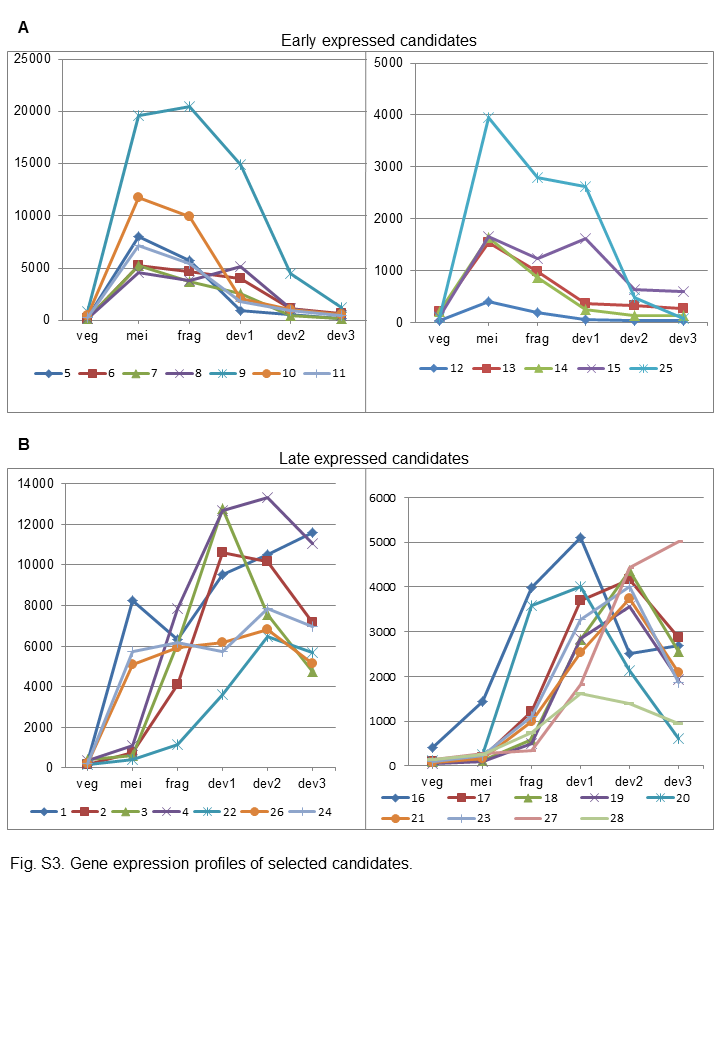

Supplement: Figure S3 — Gene expression profiles of selected candidates. (TIF) [file pone.0112899.s003.tif]

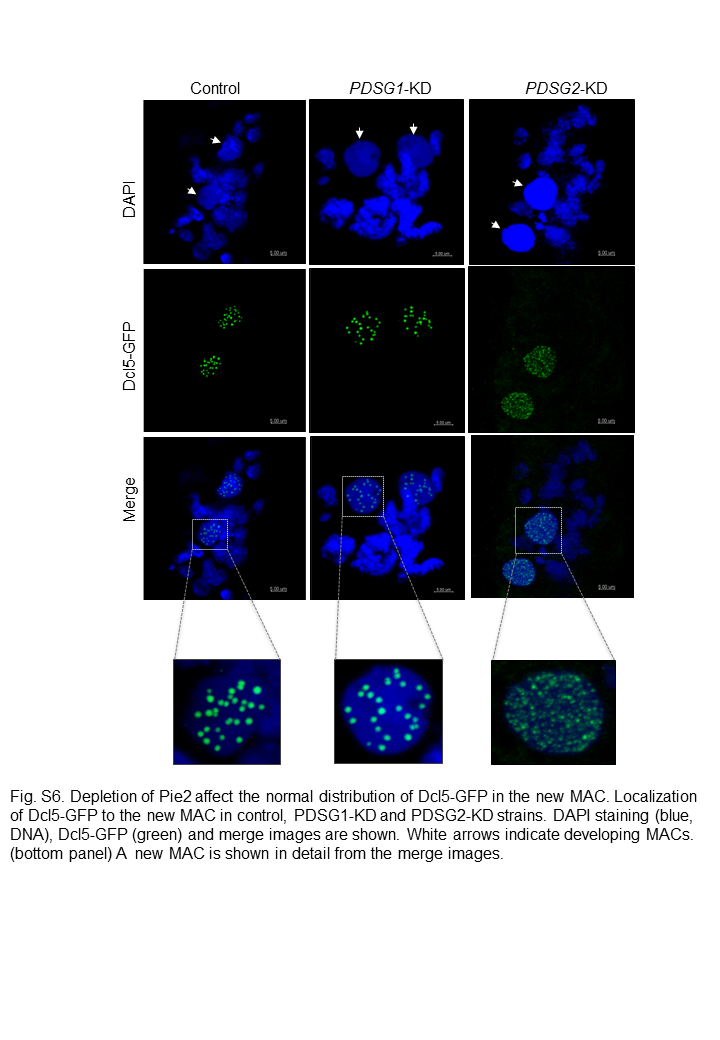

Supplement: Figure S6 — Depletion of Pdsg2 affect the normal distribution of Dcl5-GFP in the new MAC. Localization of Dcl5-GFP to the new MAC in control, PDSG1-KD and PDSG2-KD strains. DAPI staining (blue, DNA), Dcl5-GFP (green) and merge images are shown. White arrows indicate developing MACs. (bottom panel) New MAC is shown in detail from the merge images. (TIF) [file pone.0112899.s006.tif]

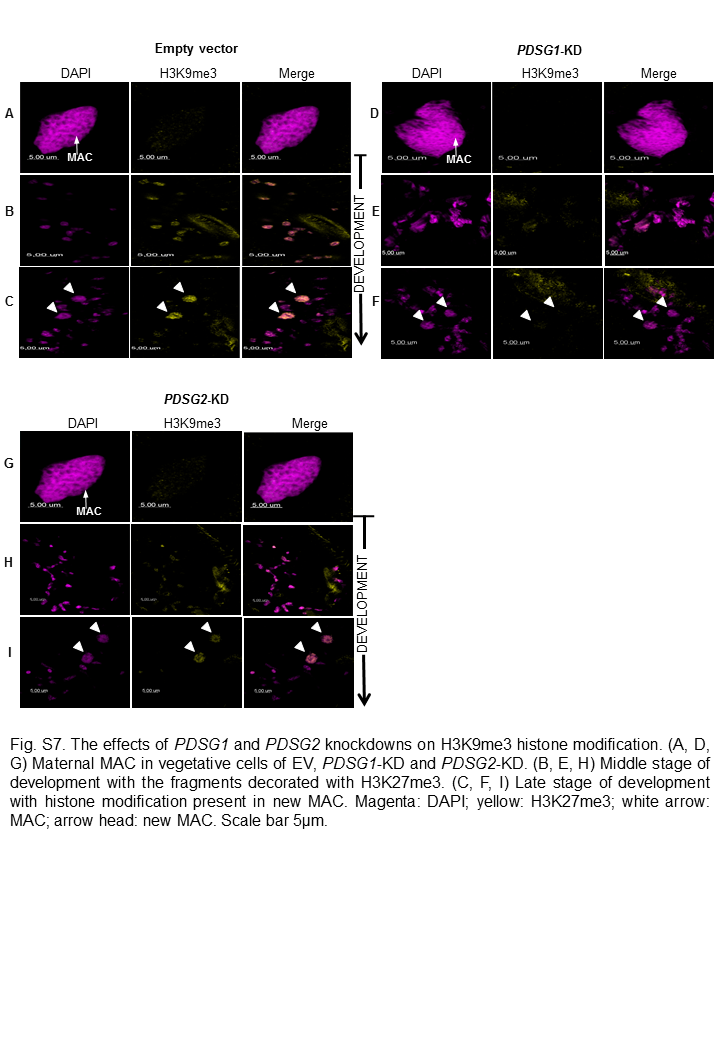

Supplement: Figure S7 — The effects of PDSG1 and PDSG2 knockdowns on H3K9me3 histone modification. (A, D, G) Maternal MAC in vegetative cells of EV, PDSG1-KD and PDSG2-KD. (B, E, H) Middle stage of development with the fragments decorated with H3K27me3. (C, F, I) Late stage of development with histone modification present in new MAC. Magenta: DAPI; yellow: H3K27me3; white arrow: MAC; arrow head: new MAC. Scale bar 5 µm. (TIF) [file pone.0112899.s007.tif]
